# Bis(3-methyl­piperidinium) naphthalene-1,5-disulfonate

**DOI:** 10.1107/S160053681202003X

**Published:** 2012-05-12

**Authors:** Qian Xu

**Affiliations:** a Ordered Matter Science Research Center, College of Chemistry and Chemical Engineering, Southeast University, Nanjing 211189, People’s Republic of China

## Abstract

The asymmetric unit of the title compound, 2C_6_H_14_N^+^·C_10_H_6_O_6_S_2_^2−^, contains one 3-methyl­piperidinium cation and one-half of the centrosymmetric naphthalene-1,5-disulfonate anion. In the crystal, anions and cations are linked through N—H⋯O hydrogen bonds into layers parallel to (101).

## Related literature

The crystal structure of the related bis­(2-methyl­piperidinium) penta­chloridoanti­monate(III) has been reported by Xu (2012 ▶).
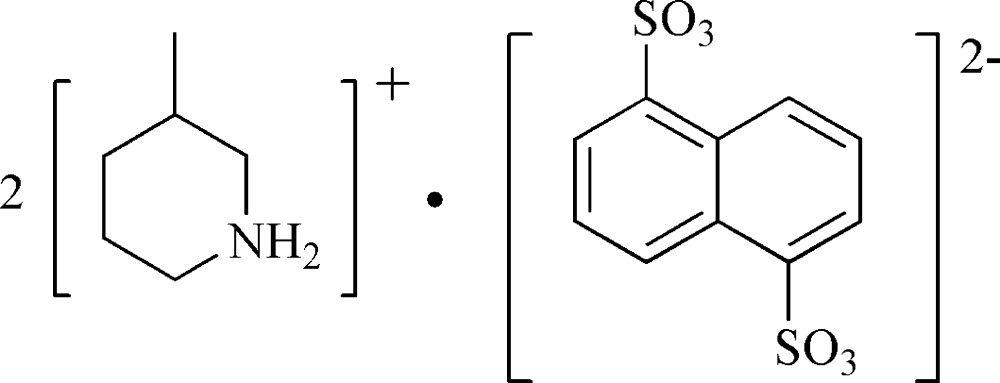


## Experimental

### 

#### Crystal data

2C_6_H_14_N^+^·C_10_H_6_O_6_S_2_^2−^
*M* *_r_* = 486.63
Monoclinic, 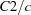

*a* = 18.100 (4) Å
*b* = 9.1763 (18) Å
*c* = 15.151 (3) Å
β = 102.06 (3)°
*V* = 2460.9 (8) Å^3^
*Z* = 4
Mo *K*α radiation
μ = 0.26 mm^−1^
*T* = 293 K
0.34 × 0.27 × 0.22 mm

#### Data collection

Rigaku Mercury70 CCD diffractometer
Absorption correction: multi-scan (*CrystalClear*; Rigaku, 2005 ▶) *T*_min_ = 0.965, *T*_max_ = 0.993
12253 measured reflections
2816 independent reflections
1835 reflections with *I* > 2σ(*I*)
*R*_int_ = 0.051

#### Refinement

*R*[*F* ^2^ > 2σ(*F* ^2^)] = 0.057
*wR*(*F* ^2^) = 0.151
*S* = 1.03
2816 reflections
146 parameters
H-atom parameters constrained
Δρ_max_ = 0.65 e Å^−3^
Δρ_min_ = −0.28 e Å^−3^

### 

Data collection: *SCXmini* (Rigaku, 2006 ▶); cell refinement: *SCXmini*; data reduction: *SCXmini*; program(s) used to solve structure: *SHELXS97* (Sheldrick, 2008 ▶); program(s) used to refine structure: *SHELXL97* (Sheldrick, 2008 ▶); molecular graphics: *DIAMOND* (Brandenburg & Putz, 2005 ▶); software used to prepare material for publication: *SHELXL97*.

## Supplementary Material

Crystal structure: contains datablock(s) I, global. DOI: 10.1107/S160053681202003X/cv5290sup1.cif

Structure factors: contains datablock(s) I. DOI: 10.1107/S160053681202003X/cv5290Isup2.hkl

Supplementary material file. DOI: 10.1107/S160053681202003X/cv5290Isup3.cml

Additional supplementary materials:  crystallographic information; 3D view; checkCIF report

## Figures and Tables

**Table 1:** Hydrogen-bond geometry (Å, °)

*D*—H⋯*A*	*D*—H	H⋯*A*	*D*⋯*A*	*D*—H⋯*A*
N1—H1*C*⋯O2	0.90	2.01	2.855 (3)	156
N1—H1*D*⋯O1^i^	0.90	1.91	2.804 (3)	175

Symmetry code: (i) 
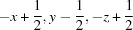
.

**Fig. 1.**
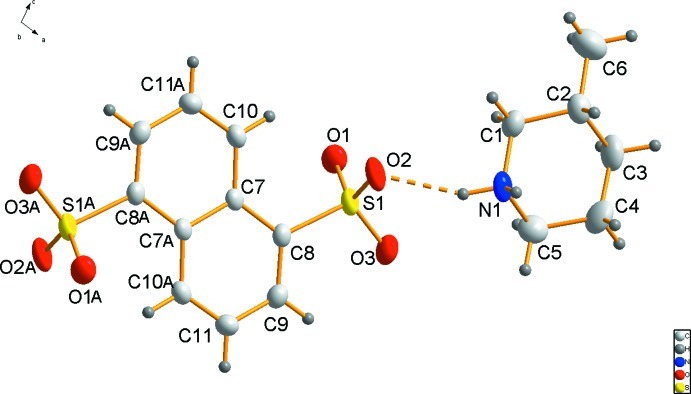
The molecular structure of (I) showing the atomic numbering and displacement ellipsoids drawn at the 30% probability level [symmetry code: (A) -x, 1 - y, -z]. Dashed line denotes hydrogen bond.

**Fig. 2.**
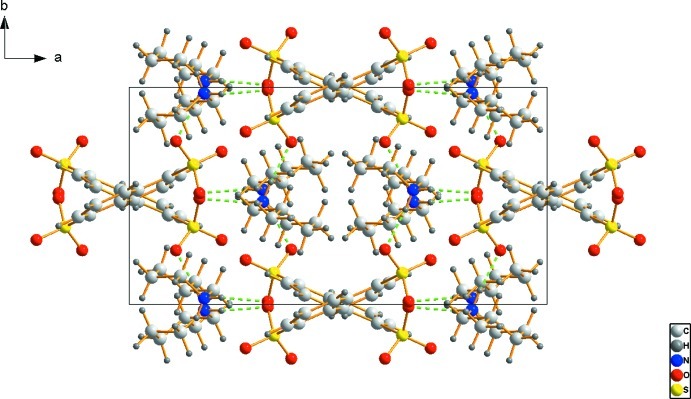
A portion of the crystal packing viewed down the c axis. Hydrogen bonds are shown as dashed lines.

## supplementary crystallographic information

### Comment

In a continuation of a structural study of new potent ferroelectric materials containing methylpiperidinium cations (Xu, 2012), we present here the title compound, (I).

The asymmetric unit of (I) contains one 3-methylpiperidinium cation and one-half of the centrosymmetric naphthalene-1,5-disulfonate anion (Fig. 1). Intermolecular N—H···O hydrogen bonds (Table 1, Fig. 2) link anions and cations into layers parallel to (101).

### Experimental

A mixture of 3-methylpiperdine (0.98 g, 10 mmol), 1,5-naphthalenedisulfonic acid (2.5 g, 10 mmol) in a water was stirred for several days at ambient temperature to obtain colourless crystals.

### Refinement

H atoms were geometrically positioned (C—H 0.93–0.97 Å; N—H 0.90 Å), and refined as riding, with *U*_iso_(H)=1.2–1.5 *U*_eq_(C, N).

### Crystal data

**Table:**

2C_6_H_14_N^+^·C_10_H_6_O_6_S_2_^2^^−^	*F*(000) = 1040
*M**_r_* = 486.63	*D*_x_ = 1.313 Mg m^−^^3^
Monoclinic, *C*2/*c*	Mo *K*α radiation, λ = 0.71073 Å
Hall symbol: -C 2yc	Cell parameters from 2816 reflections
*a* = 18.100 (4) Å	θ = 3.1–27.5°
*b* = 9.1763 (18) Å	µ = 0.26 mm^−^^1^
*c* = 15.151 (3) Å	*T* = 293 K
β = 102.06 (3)°	Prism, colourless
*V* = 2460.9 (8) Å^3^	0.34 × 0.27 × 0.22 mm
*Z* = 4	

### Data collection

**Table:**

Rigaku Mercury70 CCD diffractometer	2816 independent reflections
Radiation source: fine-focus sealed tube	1835 reflections with *I* > 2σ(*I*)
Graphite monochromator	*R*_int_ = 0.051
ω scans	θ_max_ = 27.5°, θ_min_ = 3.2°
Absorption correction: multi-scan (*CrystalClear*; Rigaku, 2005)	*h* = −22→23
*T*_min_ = 0.965, *T*_max_ = 0.993	*k* = −11→11
12253 measured reflections	*l* = −19→19

### Refinement

**Table:**

Refinement on *F*^2^	Primary atom site location: structure-invariant direct methods
Least-squares matrix: full	Secondary atom site location: difference Fourier map
*R*[*F*^2^ > 2σ(*F*^2^)] = 0.057	Hydrogen site location: inferred from neighbouring sites
*wR*(*F*^2^) = 0.151	H-atom parameters constrained
*S* = 1.03	*w* = 1/[σ^2^(*F*_o_^2^) + (0.0675*P*)^2^ + 2.1939*P*] where *P* = (*F*_o_^2^ + 2*F*_c_^2^)/3
2816 reflections	(Δ/σ)_max_ < 0.001
146 parameters	Δρ_max_ = 0.65 e Å^−^^3^
0 restraints	Δρ_min_ = −0.28 e Å^−^^3^

### Special details

**Table:**

Geometry. All e.s.d.'s (except the e.s.d. in the dihedral angle between two l.s. planes) are estimated using the full covariance matrix. The cell e.s.d.'s are taken into account individually in the estimation of e.s.d.'s in distances, angles and torsion angles; correlations between e.s.d.'s in cell parameters are only used when they are defined by crystal symmetry. An approximate (isotropic) treatment of cell e.s.d.'s is used for estimating e.s.d.'s involving l.s. planes.
Refinement. Refinement of *F*^2^ against ALL reflections. The weighted *R*-factor *wR* and goodness of fit *S* are based on *F*^2^, conventional *R*-factors *R* are based on *F*, with *F* set to zero for negative *F*^2^. The threshold expression of *F*^2^ > σ(*F*^2^) is used only for calculating *R*-factors(gt) *etc*. and is not relevant to the choice of reflections for refinement. *R*-factors based on *F*^2^ are statistically about twice as large as those based on *F*, and *R*- factors based on ALL data will be even larger.

### Fractional atomic coordinates and isotropic or equivalent isotropic displacement parameters (Å^2^)

**Table:**

	*x*	*y*	*z*	*U*_iso_*/*U*_eq_	
C1	0.29821 (16)	0.5641 (4)	0.3876 (2)	0.0640 (8)	
H1A	0.2607	0.4947	0.3987	0.077*	
H1B	0.2754	0.6602	0.3829	0.077*	
C2	0.36527 (17)	0.5617 (4)	0.4661 (2)	0.0627 (8)	
H2	0.3851	0.4620	0.4727	0.075*	
C3	0.42612 (18)	0.6595 (4)	0.4453 (3)	0.0757 (10)	
H3A	0.4710	0.6497	0.4927	0.091*	
H3B	0.4094	0.7600	0.4449	0.091*	
C4	0.44594 (19)	0.6248 (5)	0.3555 (3)	0.0841 (11)	
H4A	0.4828	0.6947	0.3434	0.101*	
H4B	0.4685	0.5285	0.3581	0.101*	
C5	0.3770 (2)	0.6296 (4)	0.2807 (3)	0.0745 (10)	
H5A	0.3566	0.7277	0.2745	0.089*	
H5B	0.3902	0.6023	0.2241	0.089*	
C6	0.3419 (3)	0.6035 (6)	0.5532 (3)	0.1106 (15)	
H6A	0.3198	0.6990	0.5471	0.166*	
H6B	0.3854	0.6033	0.6018	0.166*	
H6C	0.3055	0.5345	0.5657	0.166*	
C7	0.02115 (13)	0.5265 (3)	0.04203 (16)	0.0363 (6)	
C8	0.09422 (13)	0.5875 (3)	0.04328 (17)	0.0401 (6)	
C9	0.12180 (15)	0.5968 (3)	−0.03345 (19)	0.0491 (7)	
H9	0.1687	0.6394	−0.0316	0.059*	
C10	−0.01080 (15)	0.5181 (3)	0.11938 (18)	0.0463 (7)	
H10	0.0160	0.5547	0.1740	0.056*	
C11	0.08005 (16)	0.5426 (3)	−0.11543 (19)	0.0538 (7)	
H11	0.0998	0.5482	−0.1673	0.065*	
N1	0.32008 (12)	0.5276 (3)	0.30185 (17)	0.0556 (7)	
H1C	0.2788	0.5298	0.2569	0.067*	
H1D	0.3388	0.4365	0.3052	0.067*	
O1	0.11209 (11)	0.7517 (2)	0.18646 (14)	0.0616 (6)	
O2	0.16835 (11)	0.5125 (2)	0.20070 (14)	0.0660 (6)	
O3	0.22131 (11)	0.7037 (2)	0.12446 (15)	0.0667 (6)	
S1	0.15360 (4)	0.64375 (7)	0.14678 (5)	0.0470 (2)	

### Atomic displacement parameters (Å^2^)

**Table:**

	*U*^11^	*U*^22^	*U*^33^	*U*^12^	*U*^13^	*U*^23^
C1	0.0435 (16)	0.075 (2)	0.071 (2)	−0.0021 (15)	0.0072 (15)	−0.0047 (18)
C2	0.0613 (19)	0.0546 (18)	0.066 (2)	−0.0005 (15)	−0.0021 (16)	−0.0012 (16)
C3	0.063 (2)	0.060 (2)	0.091 (3)	−0.0163 (16)	−0.0146 (18)	−0.0074 (18)
C4	0.057 (2)	0.083 (3)	0.112 (3)	−0.0254 (19)	0.016 (2)	−0.007 (2)
C5	0.086 (2)	0.066 (2)	0.072 (2)	−0.0020 (19)	0.0185 (19)	0.0072 (18)
C6	0.129 (4)	0.130 (4)	0.072 (3)	0.000 (3)	0.019 (3)	−0.005 (3)
C7	0.0346 (12)	0.0353 (13)	0.0356 (13)	0.0082 (10)	−0.0003 (10)	0.0027 (10)
C8	0.0342 (12)	0.0392 (13)	0.0436 (15)	0.0049 (10)	0.0003 (11)	0.0013 (11)
C9	0.0373 (14)	0.0532 (17)	0.0561 (17)	0.0006 (12)	0.0083 (12)	0.0004 (13)
C10	0.0438 (14)	0.0526 (16)	0.0386 (14)	0.0018 (12)	−0.0001 (11)	0.0003 (12)
C11	0.0509 (16)	0.069 (2)	0.0421 (15)	0.0000 (14)	0.0120 (13)	0.0001 (14)
N1	0.0452 (13)	0.0484 (13)	0.0630 (15)	0.0077 (11)	−0.0122 (12)	−0.0001 (12)
O1	0.0608 (12)	0.0539 (12)	0.0676 (13)	−0.0040 (10)	0.0075 (11)	−0.0187 (10)
O2	0.0629 (13)	0.0571 (13)	0.0623 (13)	0.0007 (10)	−0.0233 (10)	0.0095 (10)
O3	0.0442 (11)	0.0723 (14)	0.0791 (15)	−0.0137 (10)	0.0026 (10)	−0.0100 (12)
S1	0.0399 (4)	0.0442 (4)	0.0493 (4)	−0.0003 (3)	−0.0077 (3)	−0.0029 (3)

### Geometric parameters (Å, º)

**Table:**

C1—N1	1.474 (4)	C6—H6C	0.9600
C1—C2	1.512 (4)	C7—C10	1.413 (4)
C1—H1A	0.9700	C7—C7^i^	1.427 (5)
C1—H1B	0.9700	C7—C8	1.433 (3)
C2—C3	1.504 (4)	C8—C9	1.360 (4)
C2—C6	1.517 (5)	C8—S1	1.782 (3)
C2—H2	0.9800	C9—C11	1.403 (4)
C3—C4	1.512 (5)	C9—H9	0.9300
C3—H3A	0.9700	C10—C11^i^	1.361 (4)
C3—H3B	0.9700	C10—H10	0.9300
C4—C5	1.501 (5)	C11—C10^i^	1.361 (4)
C4—H4A	0.9700	C11—H11	0.9300
C4—H4B	0.9700	N1—H1C	0.9000
C5—N1	1.476 (4)	N1—H1D	0.9000
C5—H5A	0.9700	O1—S1	1.447 (2)
C5—H5B	0.9700	O2—S1	1.449 (2)
C6—H6A	0.9600	O3—S1	1.447 (2)
C6—H6B	0.9600		
			
N1—C1—C2	111.8 (2)	H6A—C6—H6B	109.5
N1—C1—H1A	109.3	C2—C6—H6C	109.5
C2—C1—H1A	109.3	H6A—C6—H6C	109.5
N1—C1—H1B	109.3	H6B—C6—H6C	109.5
C2—C1—H1B	109.3	C10—C7—C7^i^	119.0 (3)
H1A—C1—H1B	107.9	C10—C7—C8	123.1 (2)
C3—C2—C6	112.4 (3)	C7^i^—C7—C8	117.8 (3)
C3—C2—C1	109.3 (3)	C9—C8—C7	121.0 (2)
C6—C2—C1	110.9 (3)	C9—C8—S1	118.2 (2)
C3—C2—H2	108.1	C7—C8—S1	120.68 (19)
C6—C2—H2	108.1	C8—C9—C11	120.6 (2)
C1—C2—H2	108.1	C8—C9—H9	119.7
C2—C3—C4	112.6 (3)	C11—C9—H9	119.7
C2—C3—H3A	109.1	C11^i^—C10—C7	121.2 (2)
C4—C3—H3A	109.1	C11^i^—C10—H10	119.4
C2—C3—H3B	109.1	C7—C10—H10	119.4
C4—C3—H3B	109.1	C10^i^—C11—C9	120.3 (3)
H3A—C3—H3B	107.8	C10^i^—C11—H11	119.8
C5—C4—C3	110.9 (3)	C9—C11—H11	119.8
C5—C4—H4A	109.5	C1—N1—C5	112.1 (3)
C3—C4—H4A	109.5	C1—N1—H1C	109.2
C5—C4—H4B	109.5	C5—N1—H1C	109.2
C3—C4—H4B	109.5	C1—N1—H1D	109.2
H4A—C4—H4B	108.0	C5—N1—H1D	109.2
N1—C5—C4	109.0 (3)	H1C—N1—H1D	107.9
N1—C5—H5A	109.9	O3—S1—O1	112.05 (13)
C4—C5—H5A	109.9	O3—S1—O2	112.52 (13)
N1—C5—H5B	109.9	O1—S1—O2	112.66 (14)
C4—C5—H5B	109.9	O3—S1—C8	106.81 (13)
H5A—C5—H5B	108.3	O1—S1—C8	107.07 (12)
C2—C6—H6A	109.5	O2—S1—C8	105.17 (12)
C2—C6—H6B	109.5		

Symmetry code: (i) −*x*, −*y*+1, −*z*.

### Hydrogen-bond geometry (Å, º)

**Table:**

*D*—H···*A*	*D*—H	H···*A*	*D*···*A*	*D*—H···*A*
N1—H1*C*···O2	0.90	2.01	2.855 (3)	156
N1—H1*D*···O1^ii^	0.90	1.91	2.804 (3)	175

Symmetry code: (ii) −*x*+1/2, *y*−1/2, −*z*+1/2.
